# Smooth Endoplasmic Reticulum Clusters in Oocytes From Patients Who Received Intracytoplasmic Sperm Injections Negatively Affect Blastocyst Quality and Speed of Blastocyst Development

**DOI:** 10.3389/fphys.2021.732547

**Published:** 2021-12-09

**Authors:** Xue Wang, YaLing Xiao, ZhengYi Sun, JingRan Zhen, Qi Yu

**Affiliations:** Department of Gynecology Endocrine and Reproductive Center, Peking Union Medical College Hospital, Peking Union Medical College, Chinese Academy of Medical Sciences, Beijing, China

**Keywords:** oocyte, smooth endoplasmic reticulum cluster, embryonic development, blastocyst formation rate, blastocyst quality

## Abstract

Findings regarding the relationship between smooth endoplasmic reticulum clusters (SERCs) in oocytes and blastocyst development have been conflicting. In this study, the effects of SERCs on blastocyst quality and the speed of blastocyst development were evaluated. Patients who received intracytoplasmic sperm injections (ICSI) at our reproductive center from 2016 to 2020 were retrospectively analyzed. SERC (+) oocytes (*n* = 217) and SERC (–) oocytes (*n* = 822), as well as SERC (+) cycles (*n* = 146) and SERC (–) cycles (*n* = 1,951) were compared. There was no significant difference in embryological, clinical, and neonatal outcomes between the SERC (+) and SERC (–) cycles. The fertilization rate (73.9%), good quality blastocyst rate (26.7%) and the speed of blastocyst development (44.4%) were significantly lower (*P* < 0.05) in SERC (+) oocytes than in unaffected counterparts (86.2%, 44.1% and 63.4%, respectively). Furthermore, the proportion of blastocysts with trophectoderm (TE) grade C was significantly higher in the SERC (+) oocyte group than in the SERC (–) oocyte group (73.3 vs. 55.9%, *P* < 0.05). After adjusting for age, years of infertility, endometriosis, stimulation protocols (GnRHa), and male infertility, multiple logistic regression analysis revealed that the presence of SERCs in the oocytes significantly affected the speed of blastocyst development (odds ratio, 2.812; 95% CI, 1.257–6.292; *P* = 0.012). These findings suggest that the presence of SERCs in oocytes may negatively affect blastocyst quality and the speed of blastocyst development.

## Introduction

Prolonging embryo culture *in vitro* to the blastocyst stage is conducive to the selection of viable embryos and thereby improves implantation rates (Glujovsky et al., 2016). Blastocysts cross the developmental block at the eight-cell stage of *in vitro* development. At this stage, the synchronization of embryonic development and endometrial changes is more conducive to blastocyst transfer (Shapiro et al., 2008), and the reduced contractile force of the uterine wall is conducive to implantation (Fanchin et al., 2001). However, blastocyst cultures have some limitations; for example, the blastocyst formation rate is unpredictable, and it may not be possible to obtain blastocysts from some patients. Although blastocyst formation is affected by many factors, obtaining good quality oocytes is critical (Wilding et al., 2007; Catalá et al., 2012). Most oocytes are evaluated based on non-invasive morphological observations, including the state of the cytoplasm and nucleus (ALPHA Scientists in Reproductive Medicine and ESHRE Special Interest Group Embryology, 2011). When assisted reproductive technologies (ART) are used, some oocytes (obtained by controlled ovarian stimulation) might exhibit morphological abnormalities, including extracytoplasmic abnormalities, such as those in polar body morphology, perivitelline space, and zona pellucida, as well as cytoplasmic abnormalities, such as abnormal refractors, intracellular particles, vacuoles, and smooth endoplasmic reticulum clusters (SERCs) (ALPHA Scientists in Reproductive Medicine and ESHRE Special Interest Group Embryology, 2011).

Under normal conditions, the oocytes contain smooth endoplasmic reticulum vesicles that can store and release calcium ions; the vesicles are also responsible for cell activation during fertilization. Calcium ions contribute to early embryonic development by regulating the stress response of oocytes or embryos and the meiotic spindle (De Santis et al., 2015; Latham, 2015, 2016). However, in some cases, the smooth endoplasmic reticulum aggregates to generate SERCs, forming a single large aggregate (Van Blerkom and Henry, 1992) presenting as a round, flat, and clear disk with a diameter of approximately 10–18 μm (Shaw-Jackson et al., 2014). SERCs is considered a major oocyte abnormality (ALPHA Scientists in Reproductive Medicine and ESHRE Special Interest Group Embryology, 2011), which can interfere with calcium storage and oscillation and may affect fertilization, embryonic development (Ebner et al., 2008; Sá et al., 2011; de Almeida Ferreira Braga et al., 2013; Mateizel et al., 2013; Sfontouris et al., 2018), and, even, birth outcomes. These findings suggest that SERC-containing oocytes should be discarded (ALPHA Scientists in Reproductive Medicine and ESHRE Special Interest Group Embryology, 2011). However, other studies have shown that the presence of SERCs does not affect the ability of oocytes to undergo embryonic development (Setti et al., 2016; Itoi et al., 2017) or to produce healthy offspring (Hattori et al., 2014; Shaw-Jackson et al., 2016; Ferreux et al., 2019). It has also been suggested that SERC (+) oocytes can avoid abnormal calcium oscillations and develop into blastocysts and that embryos derived from SERC (+) oocytes could be transferred after blastocyst culture (Itoi et al., 2017). Consequently, despite studies on the effects of SERCs on blastocyst formation and quality (de Almeida Ferreira Braga et al., 2013; Itoi et al., 2017), it is not yet clear whether the presence of SERCs in oocytes affects blastocyst formation and the data on their effect on the speed of blastocyst development are limited. Therefore, the purpose of the study was to evaluate the effects of SERCs on the quality and speed of blastocyst development.

## Materials and Methods

### Patients and Definitions

**FIGURE 1:**
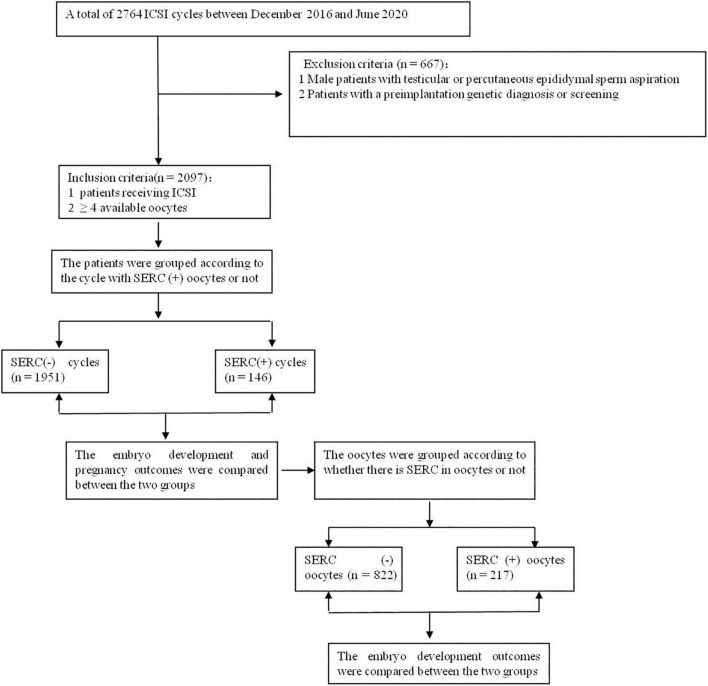
Flow chart of inclusion of patients in this study.

The retrospective study included patients who received intracytoplasmic sperm injections (ICSI) at the reproductive center of Peking Union Medical College Hospital between December 2016 and June 2020. The inclusion criteria were as follows: (1) patients receiving ICSI and (2) patients with ≥4 oocytes. Cycles with testicular or epididymal sperm aspiration or with a preimplantation genetic diagnosis or screening were excluded. SERC (+) oocytes were defined as those in which SERCs were observed by microscopy and SERC (–) oocytes were those in which SERCs were not observed. A cycle with SERC (+) oocytes was defined as a SERC (+) cycle and a cycle without SERC (+) oocytes was defined as a SERC (–) cycle, as shown in Figure 1. All patients signed a written, informed consent form, and this study was approved by the Ethics Committee of Peking Union Medical College Hospital in China (ZS-1214).

### Ovarian Stimulation and Oocyte Retrieval

Ovarian stimulation was performed using two protocols, namely, the GnRH agonist and flexible GnRH antagonist protocols. Briefly, for the GnRH agonist protocol, the patients were administered a GnRH agonist (Decapeptyl; Ferring, Germany) in the midluteal phase of the menstrual cycle for pituitary downregulation. When the downregulation was achieved, ovarian stimulation was performed using recombinant follicle-stimulating hormone (FSH, Gonal F; Merck Schlano, Switzerland) according to patients’ age, antral follicle count, and body mass index. For the antagonist protocol, ovarian stimulation was started from day 2 of the menstrual cycle with FSH (Gonal F). When dominant follicles reached a diameter of 14 mm, GnRH antagonist (Cetrotide; Merck Serono, Germany) administration was started and continued until the day of human chorionic gonadotropin (HCG; Merck Schlano) injection. In both ovarian stimulation protocols, when three or more follicles reached a diameter of 18 mm (as determined by ultrasound scanning), 250 μg of HCG (Merck Schlano) was injected for final oocyte maturation. Transvaginal oocyte retrieval was performed 38 h after HCG administration.

### Evaluation of Oocytes

**FIGURE 2:**
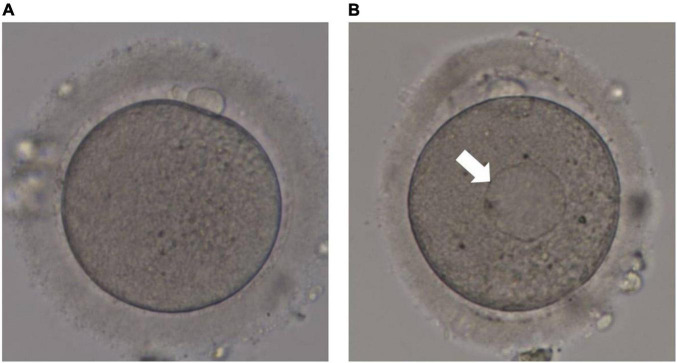
Human metaphase II oocytes as viewed by light microscopy. **(A)** Normal metaphase II oocyte; **(B)** metaphase II oocyte containing a smooth endoplasmic reticulum cluster (arrow).

The retrieved cumulus-oocyte complexes (COCs) were incubated in pre-equilibrated fertilization medium (G-IVF; Vitrolife, Sweden) at 37°C under a controlled atmosphere of 6% CO_2_ and 5% O_2_ in an incubator for 2 h until oocyte denudation. The time between HCG injection and oocyte denudation was approximately 40 h. The COCs were transferred into a medium with 80 IU/mL hyaluronidase (Sigma, United States) to disperse the cumulus cells, which were then carefully removed using a denuding Pasteur pipette with decreasing inner diameters (170–140 μm). The morphology of each oocyte was evaluated under an inverted microscope (Eclipse TE 300; Nikon) before ICSI. At high magnifications, SERCs in the cytoplasm appeared round, flat, and clear (Figure 2). Sperm injection into the aggregate was avoided during the ICSI procedure.

### Embryo Culture and Evaluation

The embryos derived from the SERC (+)/SERC (–) oocytes were cultured under the same conditions. The injected oocytes were cultured in a pre-equilibrated culture medium (G1; Vitrolife) at 37°C under a controlled atmosphere of 6% CO_2_ and 5% O_2_ in an incubator. The prokaryotic (2PN) cells were observed 18–20 h after ICSI. Cleavage embryos were evaluated 68 ± 1 h after fertilization. Good-quality embryos were defined as embryos with eight blastomeres that were symmetrical and evenly arranged, with less than 10% fragmentation. Two embryos of the best quality were transferred on day 3 (D3), and the supernumerary embryos were subsequently cultured in a pre-equilibrated medium (G2; Vitrolife) in the same incubator under 6% CO_2_ and 5% O_2_ until day 5 (D5) or day 6 (D6). If the embryos could not be transferred (e.g., due to ovarian hyperstimulation syndrome), they were cryopreserved on D3 or cultured to D5/D6. These blastocysts were then cryopreserved in liquid N_2_. The embryos originating from the SERC (+) oocytes were only transferred if there were no other embryos to transfer. Luteal support (intramuscular progesterone 40 mg/bid or intramuscular progesterone 40 mg/d + vaginal progesterone gel 90 mg/d) was started on the day of transplantation.

Blastocysts were observed on D5 (118–120 h) or D6 (142–144 h) after fertilization and scored according to the Gardner scoring system (Gardner et al., 2000). Based on the degree of cavity expansion, blastocysts can be divided into six stages: stage 1, early blastocysts with a blastocoel that is less than half the volume of the embryo; stage 2, blastocysts with a blastocoel that is greater than half the volume of the embryo; stage 3, full blastocysts with a blastocoel that completely fills the embryo; stage 4, blastocysts show full cavity expansion and zona pellucida thinning; stage 5, blastocysts have partial trophoblasts growing from the zona pellucida; and stage 6, blastocysts are completely detached from the zona pellucida. Blastocyst quality was graded according to the quality of the inner cell mass (ICM) and trophectoderm (TE). The ICM and TE were divided into grade A (more cells, tightly packed), grade B (several cells, loosely arranged), and grade C (very few cells). Blastocysts with a grade C ICM were discarded, and blastocysts with ICM (A/B) and TE (A/B/C) were selected for freezing. Embryos with the ICM and TE qualities above grade B (AA, AB, BA, and BB) were defined as good-quality embryos and those identified as BC were defined as poor quality embryos. All available blastocysts formed on D5/D6 were cryopreserved in liquid N_2_ until the next embryo transfer.

### Outcome Parameters

The main outcome of this study was the effect of SERCs on blastocyst quality and speed of blastocyst development (compared by D5 blastocyst formation rate); the clinical and neonatal outcomes were secondary. The outcome parameters were the mature oocyte rate, fertilization rate, D3 good-quality embryo rate, blastocyst formation rate, D5 blastocyst formation rate, good quality blastocyst rate, implantation rate, clinical pregnancy rate, and live birth rate, defined as follows:

Mature oocyte rate (%) = (number of MII oocytes/total oocytes retrieved) × 100; Fertilization rate (%) = (number of fertilized oocytes/number of MII oocytes) × 100; D3 good-quality embryo rate (%) = (number of D3 good-quality embryos/number of cleavage embryos) × 100; Blastocyst formation rate (%) = [number of blastocysts/number of embryos that cultured for blastocysts] × 100; D5 blastocyst formation rate (%) = (number of blastocysts formed on D5/total number of blastocysts) × 100; Good quality blastocyst rate (%) = (total number of good quality blastocysts/total number of blastocysts) × 100; Implantation rate = number of gestational sacs/number of transferred embryos × 100; Clinical pregnancy rate = number of patients with at least one gestational sac (intra or extrauterine)/number of transferred cycles; Live birth rate = number of deliveries/number of transferred cycles.

### Statistical Analysis

Statistical analyses were performed using SPSS 22.0 (IBM, Armonk, NY, United States). Data are presented as the mean ± standard deviation, and Student’s parametric *t*-test or Mann–Whitney non-parametric tests were used to compare the mean values as appropriate. The mature oocyte rate, fertilization rate, D3 good-quality embryo rate, blastocyst formation rate, good-quality blastocyst rate, implantation rate, clinical pregnancy rate, and live birth rate were compared using chi-square or Fisher exact test. A logistic regression model was used to assess the confounder factors that could affect the speed of blastocyst development in SERC (+) cycles. Results with *P* < 0.05 were considered significant.

## Results

A total of 2097 ICSI cycles were included, of which 146 cycles showed at least one oocyte with SERC. As summarized in Table 1, demographic data were not significantly different between the SERC (+) and SERC (–) cycles. However, the ratio of GnRH agonist used in the SERC (+) cycle was significantly greater than that in the SERC (–) cycle (72.6 vs. 58.3%, *P* = 0.001). Hormone concentrations at the time of HCG administration did not show significant differences. The mature oocyte rate in the SERC (+) cycle group was significantly higher than that in the SERC (–) cycle group (88.4 vs. 86.2%, *P* = 0.035). On the contrary, the rates of fertilization, good-quality embryos on day 3, blastocyst formation, and good-quality blastocysts, and the speed of blastocyst development did not significantly differ between the SERC (+) and SERC (–) cycle groups. However, the proportion of blastocysts with TE grade A in the SERC (+) cycle group was significantly lower than that in the SERC (–) cycle group (3.9 vs. 7.6%, *P* = 0.037). In terms of pregnancy outcome, as shown in Table 2, there were no significant differences in the implantation, clinical pregnancy, abortion, or live birth rates between the SERC (+) and SERC (–) cycle groups. Regarding birth, an analysis of obstetric data revealed that the SERC (+) cycle group could achieve the same outcomes as the SERC (–) cycle group. No neonatal malformations were detected in the SERC (+) cycle group (Table 2).

**TABLE 1:** Patient characteristics in the SERC (+) and SER (–) cycle groups.

Items	SERC (+) cycles	SERC (–) cycles	*P*-value
No. of cycles	146	1,951	
Age (years)	35.3 ± 4.0	35.1 ± 4.9	0.570
Duration of sterility (years)	4.4 ± 3.0	4.8 ± 3.0	0.646
Basal FSH (IU/L)	7.9 ± 2.5	7.8 ± 2.4	0.621
Basal LH (IU/L)	4.2 ± 3.0	4.3 ± 2.4	0.562
Total dose of FSH (IU/mL)	2610 ± 510	2530 ± 810	0.421
LH on day of HCG (U/L)	2.2 ± 1.0	2.1 ± 0.8	0.461
E_2_ on day of HCG (pg/mL)	1049.9 ± 272.1	914.0 ± 286.6	0.118
P on day of HCG (ng/mL)	1.0 ± 0.4	1.1 ± 0.5	0.233
% GnRH agonist	72.6 (106/146)	58.3 (1,138/1,951)	0.001

*SERC, smooth endoplasmic reticulum cluster; FSH, follicle-stimulating hormone; LH, luteinizing hormone; E2, oestrogen; HCG, chorionic gonadotrophin; GnRH, gonadotropin-releasing hormone.*

**TABLE 2:** Embryological, clinical, and neonatal outcomes in the SERC (+) and SER (–) cycle groups.

Items	SERC (+) cycles	SERC (–) cycles	*P*-value
No. of cycles	146	1951	
No. of oocytes	8.0 ± 4.2	8.6 ± 4.3	0.117
% MII oocytes/cycle	88.4 (1039/1175)	86.2 (14,537/16,856)	0.035
Fertilization rate	83.6 (869/1039)	85.0 (12,356/14,537)	0.237
Good quality embryos rate at D3	12.8 (108/846)	11.1 (1,363/12,257)	0.142
Blastocyst formation rate	36.7 (231/630)	36.6 (3,438/9,381)	0.993
Speed of blastocyst development, D5%	59.7 (138/231)	55.1 (1,895/3,438)	0.171
Good quality blastocyst rate	40.7 (94/231)	46.4 (1,595/3,438)	0.092
**Trophoblast cell evaluation**			
Grade A	3.9 (9/231)	7.6 (261/3,438)	0.037
Grade B	36.8 (85/231)	38.8 (1,334/3,438)	0.545
Grade C	59.3 (137/231)	53.6 (1,843/3,438)	0.092
**Clinical outcomes**			
Implantation rate	23.6 (51/216)	27.5 (791/2,876)	0.215
Biochemical pregnancy rate	6.1 (7/114)	5.6 (83/1,484)	0.807
Clinical pregnancy rate	36.8 (42/114)	41.6 (618/1,484)	0.316
Miscarriage rate	14.3 (6/42)	18.6 (115/618)	0.484
**Neonatal outcomes**			
Live birth rate	31.6 (36/114)	33.9 (503/1,484)	0.614
Age of gestation (d)	267.8 ± 12.3	268.6 ± 14.8	0.731
Birth weight (g)	2978.6 ± 580.5	2902.1 ± 660.6	0.742
Average length (cm)	49.7 ± 2.3	49.3 ± 2.9	0.730
Gender (males/females)	21:20	318:298	0.960
Twin rate	13.9 (5/36)	22.5 (113/503)	0.229
Congenital abnormalities rate	0.0	0.0 (2/503)	1.000

*SERC, smooth endoplasmic reticulum cluster; D5, day 5. Speed of blastocyst development was compared by D5 blastocyst formation rate.*

The embryological outcomes were compared between SERC (+) oocytes and SERC (–) oocytes within the SERC (+) cycle group. As shown in Table 3, the fertilization rate of SERC (+) oocytes was significantly lower than that of SERC (–) oocytes (73.9 vs. 86.2%, *P* < 0.05). Although the rates of good-quality embryos on D3 and blastocyst formation were similar, the speed of blastocyst development was significantly lower in the SERC (+) oocyte group than in the SERC (–) oocyte group (44.4 vs. 63.4%, *P* = 0.02). The proportion of blastocysts with TE grade C was significantly higher in the SERC (+) oocyte group than in the SERC (–) oocyte group (73.3 vs. 55.9%, *P* = 0.033). After adjusting for age, years of infertility, endometriosis, stimulation protocols (GnRHa), and male infertility, multiple logistic regression analysis revealed that the presence of SERCs in the oocytes significantly affected the speed of blastocyst development (odds ratio [OR], 2.812; 95% CI, 1.257–6.292; *P* = 0.012) (Table 4). A total of 28 patients had at least one embryo originating from SERC (+) oocytes transferred. Nine patients had all embryos from SERC (+) oocytes transferred; two pregnancies were achieved, resulting in the birth of two healthy infants. Nineteen patients had mixed embryos transferred, leading to eight clinical pregnancies and eight healthy births (one twin pregnancy). Moreover, 86 patients had two embryos transferred, both of which were from SERC (–) oocytes, and 32 patients successfully conceived. Although the live birth rate in the SERC (+) oocyte group was slightly lower than that in the other two groups, the difference was not significant. The neonatal outcomes were similar among the three groups, as shown in Table 5.

**TABLE 3:** Comparison of embryonic outcomes between SERC (+) and SERC (–) oocytes in the SERC (+) cycle group.

Items	SERC (+) oocytes	SERC (–) oocytes	*P*-value
No. of MII oocytes	217	822	
Fertilization rate	73.7 (160/217)	86.3 (709/822)	0.000
Good quality embryos rate at day 3	12.3 (19/154)	12.9 (89/692)	0.860
Blastocyst formation rate	35.2 (45/128)	37.1 (186/502)	0.691
Speed of blastocyst development, D5%	44.4 (20/45)	63.4 (118/186)	0.020
Good quality blastocyst rate	26.7 (12/45)	44.1 (82/186)	0.033
Trophoblast cell evaluation			
Grade A	0.0	4.8 (9/186)	0.132
Grade B	26.7 (12/45)	39.2 (73/186)	0.116
Grade C	73.3 (33/45)	55.9 (104/186)	0.033

*SERC, smooth endoplasmic reticulum cluster; D5, day 5.*

**TABLE 4:** Logistic regression analysis of SERC in oocytes that may affect the speed of blastocyst development.

Variables	OR	95% CI	*P-*value
**Speed of blastocyst development**			
Age	1.071	0.952–1.206	0.254
No. of previous IVF times	1.022	0.632–1.654	0.928
Duration of sterility	0.994	0.853–1.159	0.943
Severity of male infertility	0.587	0.192–1.792	0.350
Endometriosis	0.797	0.284–2.240	0.667
Stimulation protocols (GnRHa)	1.373	0.568–3.321	0.482
SERC(+) oocytes	2.812	1.257–6.292	0.012

*SERC, smooth endoplasmic reticulum cluster; GnRHa, gonadotropin-releasing hormone agonist; SERC (+) oocytes, blastocysts resulting from SERC (+) oocytes; CI, confidence interval; OR, odds ratio. Results with P < 0.05 were considered statistically significant.*

**TABLE 5:** Clinical and neonatal outcomes in the transfer cycles with SERC (+) oocytes.

Items	SERC(+) oocytes	Mixed oocytes	SERC (–) oocytes	*P*-value
No. of transferred cycles	9	19	86	
Implantation rate	12.5 (2/16)	23.7 (9/38)	24.7 (40/162)	0.549
Biochemical pregnancy rate	22.2 (2/9)	10.5 (2/19)	3.5 (3/86)	0.057
Clinical pregnancy rate	22.2 (2/9)	42.1 (8/19)	37.2 (32/86)	0.589
Miscarriage rate	0.0	12.5 (1/8)	15.6 (5/32)	0.825
Neonatal outcomes				
Live birth rate	22.2 (2/9)	36.8 (7/19)	31.4 (27/86)	0.737
Age of gestation (d)	277.0 ± 5.7	272.9 ± 6.9	266.9 ± 12.8	0.648
Birth weight (g)	3685 ± 21.2	3045.6 ± 339.6	2943.8 ± 613.1	0.265
Average length (cm)	50.5 ± 0.7	49.8 ± 0.6	49.3 ± 1.6	0.362
Gender (males/females)	2:0	4:4	15: 16	0.366
Twin rate	0.0	14.3 (1/7)	14.8 (4/27)	0.972
Congenital abnormalities rate	0	0	0	

*SERC (+) oocytes, the embryos transferred only from SERC (+) oocytes; mixed oocytes, the embryos transferred from one SERC (–) oocyte and one SERC (+) oocyte; SERC (–) oocytes, the embryos transferred only from SERC (–) oocytes; SERC, smooth endoplasmic reticulum cluster.*

## Discussion

The main purpose of this study was to determine the effect of the presence of SERCs in oocytes on the blastocyst quality and speed of blastocyst development. To the best of our knowledge, we analyzed the relationship between SERCs and the speed of blastocyst development for the first time. Our data showed that the blastocysts derived from SERC (+) oocytes were of a low quality (with TE grade C) and presented a tendency to be formed on D6 after fertilization, than on D5. The present study found there were no significant differences in the fertilization, good-quality embryo frequency, and blastocyst formation rates between the SERC (+) and SERC (–) cycle groups which is consistent with the findings of previous studies (Hattori et al., 2014; Itoi et al., 2017). However, these results were in contrast to some findings. Sá et al. (2011) found that the fertilization, cleavage, and blastocyst formation rates were significantly reduced in the SERC (+) cycle group compared with that in the SERC (–) cycle group. Restelli et al. (2015) found that the fertilization rate in the SERC (+) cycle group was significantly lower than that in the SERC (–) cycle group; however, once the fertilization was completed, the cleavage and implantation rates were similar between the groups. Mateizel et al. (2013) observed a significantly lower blastocyst formation rate in the SERC (+) cycles than in the SERC (–) cycles, and the capacity to develop into good-quality embryos were similar between the SERC (+) oocytes and their sibling SERC (–) oocytes within the SERC (+) cycles which supported the hypothesis that the intrinsic developmental capacity of the entire group of oocytes from SERC (+) cycles, than from SERC (+) oocytes only, was reduced.

Previous investigations concluded that compared with SERC (–) oocytes, SERC (+) oocytes exhibited a poor embryonic development (Ebner et al., 2008; de Almeida Ferreira Braga et al., 2013). In this study, the fertilization rate of SERC (+) oocytes was significantly lower than that of SERC (–) oocytes, but no difference was observed in the blastocyst formation and good-quality blastocyst rates between the groups, which was consistent with the results of Hattori et al. (2014). However, Ebner et al. (2008) found that the fertilization rate (58.9%) and blastulation rate (44.0%) were lower in SERC (+) oocytes than in their unaffected counterparts (77.4 and 87.8%, respectively), with no difference in the quality of blastocysts. In the conventional IVF cycle, the presence of SERCs in oocytes did not affect the fertilization rate or cleavage embryo quality, but resulted in a lower blastocyst formation rate and lower good-quality blastocyst rate than those in the control group (Itoi et al., 2016), similar to the conclusions of de Almeida Ferreira Braga et al. (2013), who found the presence of SERCs affects blastocyst expansion and the quality of the ICM. However, the study did not identify an effect of SERCs on TE grade.

To further analyse the effect of SERCs on blastocyst quality, we attempted to group blastocysts according to the morphology of ICM and TE. As embryos with ICM grade C were discarded because of poor implantation potential, and blastocysts with ICM grade A were few, the comparisons were not based on the ICM grade but based on trophoblast cell morphology. To the best of our knowledge, we found, for the first time, that the rate of blastocysts with TE grade A in the SERC (+) cycle group was lower than that in the SERC (–) cycle group. The proportion of blastocysts with TE grade C was significantly higher in the SERC (+) oocyte group than in the SERC (–) oocyte group. Many studies have confirmed that TE morphology is positively correlated with the embryo implantation rate (Ahlström et al., 2011; Hill et al., 2013). Alfarawati et al. (2011) found that the morphology of trophoblast cells is positively correlated with the incidence of blastocyst aneuploidy. Blastocysts with TE grade C exhibited a 2.5-times higher proportion of aneuploidy than those with TE grade A. Otsuki et al. (2018) found that the error rate in mitosis or meiosis in SERC (+) oocytes was significantly higher than that in SERC (–) oocytes, which suggested that the embryos with failed cleavage are prone to accumulate chromosomal abnormalities, but the mechanism of the frequent failure of cytokinesis in embryos derived from SERC (+) oocytes is still unknown.

To the best of our knowledge, this is the first study to analyse the relationship between SERCs and the speed of blastocyst development. It is well known that some blastocysts are formed on D5, whereas others are formed on D6/D7 (Bourdon et al., 2019). In this study, our results showed that the speed of blastocyst development, shown as the blastocysts formed rate on D5, did not differ between the SERC (+) and SERC (–) cycles but was significantly lower in the SERC (+) oocyte group than in the SERC (–) oocyte group. After adjusting for possible confounding factors, SERCs in the oocytes was still an independent factor to affect the speed of blastocyst development. Numerous studies have confirmed that the implantation, clinical pregnancy, and continuous pregnancy rates of D5 blastocysts are significantly higher than those of D6 blastocysts, suggesting that faster blastocyst development is related to better clinical outcomes (Bourdon et al., 2019). This difference can be explained by the higher rates of aneuploidy and gene abnormalities in blastocysts with delayed development (Taylor et al., 2014; Kaing et al., 2018). It is possible that D5 blastocysts are more likely to be selected for blastocyst transfer among patients who do not undergo preimplantation genetic screening (Minasi et al., 2016). In addition, studies have found that the implantation rate and live birth rate are higher with D5 blastocysts than with D6 blastocysts if only euploid embryos are transferred, they thought that the difference could be attributed not only to the euploid rate, but also to additional factors, including the differences in embryo metabolism or epigenetics (Irani et al., 2018). However, why the SERC (+) oocyte-induced slow blastocyst development is still unknown, which may also be attributed to the abnormal increase in the number of blastocyst chromosomes induced by cleavage failure, metabolic reasons, or other factors. The specific mechanisms still need to be determined.

In this study, we found that there were no significant differences in the clinical and neonatal outcomes between the SERC (+) and SERC (–) cycle group, which was similar to the conclusions of Hattori et al. (2014). In addition, we found that irrespective of whether we transferred embryos from SERC oocytes or not, the neonatal outcomes were similar; this was consistent with the finding of Mateizel et al. (2013). However, some different conclusions suggested that SERC may be associated with poor clinical and neonatal outcomes, as Ebner et al. (2008) suggested that embryos derived from SERC (+) oocytes were more prone to preterm birth and a low birth weight, whereas other studies have found that the proportion of neonatal congenital abnormalities increased after the transfer of embryos derived from SERC (+) oocytes (Otsuki et al., 2004; Akarsu et al., 2009; Sá et al., 2011). A literature review showed that compared with the natural cycle, children born using ART are more likely to have imprinting defects (Manipalviratn et al., 2009), and it is difficult to determine if the imprinting defects are caused by SERCs. Moreover, the differences in findings among the studies may be because of their designs, sample sizes, ovarian stimulation methods, or population variations.

There were some limitations in this study. First, the number of patients who had embryos transferred from SERC (+) oocytes was relatively low, which may have affected the comparisons of the neonatal outcomes. In the future, a multi-center study with a large sample size should be conducted to address this issue. Second, as this was a retrospective study, we could not investigate the chromosomes of the blastocysts to determine their relationship with SERCs and blastocyst quality and speed of blastocyst development. In the future, a study involving the biopsy of blastocysts derived from SERC (+) oocytes should be conducted to address this. This would enable us to analyse whether there is a correlation between SERCs and chromosomal abnormalities of the blastocysts. Blastocysts derived from SERC (+) oocytes that are transplanted after preimplantation genetic testing for aneuploidies may have a better clinical outcome. Third, in our study, not all embryos were cultured up to the blastocyst stage, but the embryos with the best morphology were transferred at the cleavage stage (D3). Therefore, this study does not allow a complete evaluation of the effect of the presence of SERCs on the competence of oocytes to reach the blastocyst stage. It is possible that the more competent embryos were excluded from the analysis. In the future, a prospective, randomized, controlled study should be conducted to address this limitation. Finally, there was no long-term follow-up of the new-borns. As normal development at birth does not guarantee that issues may not occur in the future, a long-term follow-up study of the children from SERC (+) oocytes should be conducted to investigate whether there are abnormalities in any later developmental processes.

In conclusion, although the presence of SERCs does not affect the clinical and neonatal outcomes, it is related to the blastocyst quality and speed of blastocyst development. Most blastocysts derived from SERC (+) oocytes had grade C trophoblast cells. Furthermore, the speed of blastocyst development in SERC (+) oocytes was significantly slower, observed as a higher blastocyst formation rate on D6. However, further studies are required to determine the specific mechanisms underlying this phenomenon.

## Data Availability Statement

All datasets generated for this study are included in the article.

## Ethics Statement

The studies involving human participants were reviewed and approved by The Institutional Review Board (IRB) of Peking Union Medical College Hospital, Chinese Academy of Medical Sciences. The patients/participants provided their written informed consent to participate in this study. Written informed consent was obtained from the individual(s) for the publication of any potentially identifiable images or data included in this article.

## Author Contributions

XW, ZS, and QY: conceptualization. XW and YX: investigation. JZ and ZS: formal analysis. XW: writing – original draft and review and editing. All authors read and approved the final manuscript.

## Conflict of Interest

The authors declare that the research was conducted in the absence of any commercial or financial relationships that could be construed as a potential conflict of interest.

## Publisher’s Note

All claims expressed in this article are solely those of the authors and do not necessarily represent those of their affiliated organizations, or those of the publisher, the editors and the reviewers. Any product that may be evaluated in this article, or claim that may be made by its manufacturer, is not guaranteed or endorsed by the publisher.
